# Atomic force microscopy analysis of native infectious and inactivated SARS-CoV-2 virions

**DOI:** 10.1038/s41598-021-91371-4

**Published:** 2021-06-04

**Authors:** Sébastien Lyonnais, Mathilde Hénaut, Aymeric Neyret, Peggy Merida, Chantal Cazevieille, Nathalie Gros, Christine Chable-Bessia, Delphine Muriaux

**Affiliations:** 1 CEMIPAI, University of Montpellier, UAR3725 CNRS, Montpellier, France; 2 Institute of Research in Infectiology of Montpellier (IRIM), University of Montpellier, UMR9004 CNRS, Montpellier, France; 3 Institut des Neurosciences de Montpellier (INM), Université de Montpellier, Montpellier, France

**Keywords:** Atomic force microscopy, SARS-CoV-2

## Abstract

SARS-CoV-2 is an enveloped virus responsible for the Coronavirus Disease 2019 (COVID-19) pandemic. Here, single viruses were analyzed by atomic force microscopy (AFM) operating directly in a level 3 biosafety (BSL3) facility, which appeared as a fast and powerful method to assess at the nanoscale level and in 3D infectious virus morphology in its native conformation, or upon inactivation treatments. AFM imaging reveals structurally intact infectious and inactivated SARS-CoV-2 upon low concentration of formaldehyde treatment. This protocol combining AFM and plaque assays allows the preparation of intact inactivated SARS-CoV-2 particles for safe use of samples out of level 3 laboratory to accelerate researches against the COVID-19 pandemic. Overall, we illustrate how adapted BSL3-AFM is a remarkable toolbox for rapid and direct virus analysis based on nanoscale morphology.

## Introduction

Severe acute respiratory syndrome coronavirus 2 (SARS-CoV-2) has emerged as a new zoonotic coronavirus in late 2019 in Wuhan, China, and rapidly spread worldwide, causing a global pandemic^1,2^. SARS-CoV-2 is an enveloped single strand ( +) RNA virus with a surface layer of glycoprotein spike (S) forming a crown-like structure. The genome is packaged into a spherical, basket-like ribonucleoprotein RNP complex by the nucleocapsid protein (N)^3^. Two other structural proteins are associated with the viral envelope: membrane (M) and envelope (E). Cellular entry of SARS-CoV-2 depends on the binding of the S glycoprotein to angiotensin-converting enzyme 2 (ACE2), which is a specific cellular receptor located on the surface of the host cell^4,5^. The viral envelope has been proposed as being highly dynamic, with the hinges of S hinges allowing significant rotational movements^6,7^.

We focused here on characterizing native and inactivated SARS-CoV-2 morphology at the nanoscale using BSL3 based Atomic Force Microscopy. This virus being classified as a hazard group 3 pathogen, it must be studied and manipulated in level 3 biosafety laboratory. Therefore, well-controlled virus inactivation methods are needed to transfer virus samples from containment level 3 to a standard laboratory, to facilitate research that does not require infectious viruses. Inactivation of viral particles can be achieved in many ways, like the application of heat, alcohol, peroxide, radiation, fixatives, or detergents, and have been described for SARS-CoV-2^8,9^ and SARS-CoV^10,11^, a previous coronavirus that spread in 2002–2003. However, published experimental data that demonstrate SARS-CoV-2 inactivation keeping intact particles are lacking, which can be necessary for applications and diagnosis requiring non-infectious particles keeping a native morphology. Therefore, we investigated methods allowing the inactivation of SARS-CoV-2 virions while maintaining their morphology. Herein, we focused on formaldehyde (FA) fixation, which is well established in histology and electron microscopy^12^. Unlike glutaraldehyde, which can cause aggregates, buffered FA has been shown to preserve virus morphology^13^, and its virucidal efficiency is very well documented, e.g., in vaccination programs^14,15^. SARS-CoV-2 virions were analyzed on a customized bio-Atomic Force Microscope (Bio-AFM) operating in BSL3. Atomic force microscopy is a unique technique that enables imaging of biological samples in their native liquid environment at the molecular scale. This technique has been widely used for virus imaging^16,17^, including recent studies on SARS-CoV-2 in liquid^18^ and in air^19^. In AFM, the sample does not require to be fixed, coated, or stained, which allowed us to image at high resolution, in 3D, native and inactivated hydrated viral particles in buffer, without any further treatment of the sample. As a result, we identified conditions for FA-based inactivation of SARS-CoV2 particles which maintain their physical integrity, while being non-infectious. Moreover, we demonstrate that modern bio-AFM is of great power for probing the structure of native and inactivated viruses at the nanoscale within minutes.

## Results

**Figure 1:**
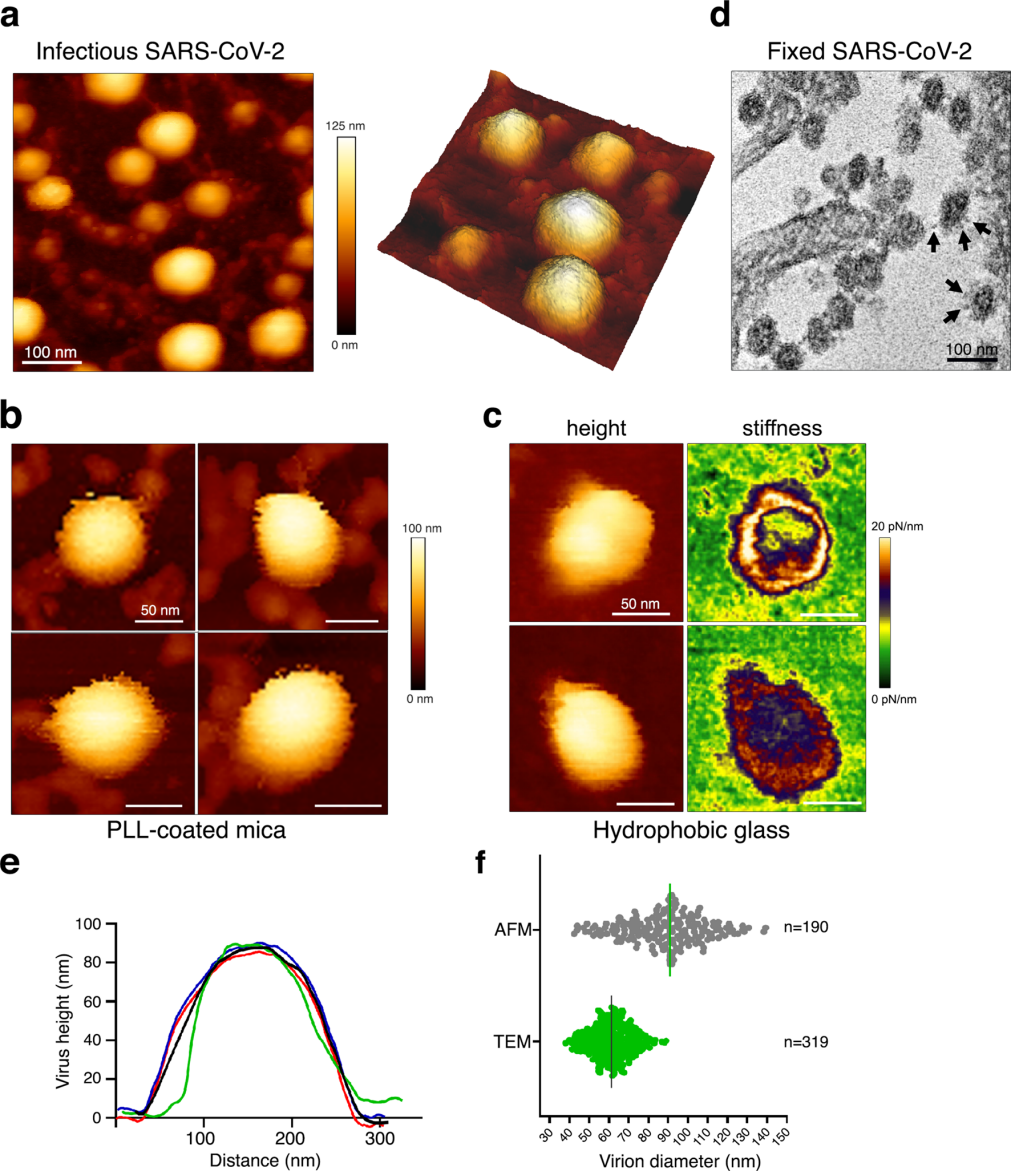
Native infectious SARS-CoV-2 virions imaged by AFM **(a)** Topographic image and 3D projection of native infectious SARS-CoV-2 virions adsorbed on a poly-l-lysine (PLL)-coated mica surface using quantitative imaging (QI) mode AFM in buffer. (**b,c**) Zoom-in view of SARS-CoV-2 virions adsorbed on poly-l-lysine-coated mica (**b**) or glass coverslips coated with an alkyl silane (**c**) (scale bars: 50 nm). In (**c**) height and stiffness images acquired in QI mode on individual virions are shown. (**d**) Example of a TEM image of fixed infected VeroE6 cells producing SARS-CoV-2 that can be seen as spherical particles studded with S trimers (see arrows). (**e**) Examples of topographical profile plots measured along the horizontal diameter of viral particles. (**f**) Virion diameter distribution of infectious SARS-CoV2 samples imaged by AFM in liquid (height profile, n = 190 objects) and compared to fixed samples imaged by TEM (n = 319 objects).

We first controlled that the virus isolate was SARS-CoV-2 by revealing the presence of the M, N, and S viral proteins in infected cell lysates using immunoblots, by quantitative RT-PCR targeting the E gene overtime on the cell culture supernatant, and by imaging fixed infected cells by Transmission Electron Microscopy (TEM) (Supplementary Figure S1). One can see that the infected cells are producing SARS-CoV-2. Native infectious SARS-CoV-2 from the clarified infected cell supernatant were then directly visualized by AFM using a force-curve based imaging mode, after smooth adsorption of the sample on an appropriate AFM surface (mica or glass coverslip). Viruses did not adsorb on freshly cleaved mica or glass coverslip (not shown), but were found to bind very quickly (2–3 min) on poly-l-lysine coated mica or glass coverslips coated with an alkyl silane to make them hydrophobic^20^. Virus adsorption on poly-l-lysine coated mica surface showed SARS-CoV-2 virions as roughly spherical or ellipsoidal particles (Fig. 1), consistent with previous electron microscopy and AFM studies. Surprisingly, a fraction of the viral particles appeared embedded in a network of thin filamentous structures adhering to the surface (Fig. 1a and Supplementary Figure S2), in addition to free particles, suggesting that SARS-CoV-2 particles could be released from the infected culture cells as viral “packages” containing tethered particles. As seen by Kiss et al., the AFM images showed “bald” native viral particles with a blurred, smooth surface, without clear S trimers protruding from the viral surface, even from viruses directly imaged from cell supernatant. This has been attributed to the high mobility of spikes and their rapid motion on virus surface, which thus evade the AFM cantilever tip^18^. We also consider that virion movements on the mica surface during adsorption could break the long spike proteins. Virions adsorbed on the hydrophobic surface showed equivalent topographical structure and the method did not help to resolve S trimers (Fig. 1c). On this surface, however, images based on the slope of the force-distance curve for an applied force of 1 nN showed particles with a donut-shape, hollowed out in the center and displaying a crown of increased stiffness in the range of 10–20 pN/nm (Fig. 1c), similar to the stiffness measured by indentation^18^. This supports the hypothesis that SARS-CoV-2 is easily deformable and that the ribonucleoprotein complex, made of N and the viral RNA, contributes little to the viral mechanics. TEM observation of the virus-producing cells confirmed unambiguously the typical structure of coronavirus particles, containing granular densities corresponding to the viral nucleoprotein and showing protruding spikes S proteins incorporated into the viral lipid envelope^22^ (Fig. 1d). The mean central height of the virions (Fig. 1e) ranged from 45 to 140 nm on both surfaces, with a mean height of 89 nm ± 19 nm (Fig. 1f), consistent with viral particle diameters in the literature measured by AFM^18^ and very close from the 91 ± 11 nm value obtained by cryo-electron tomography for SARS-CoV-2^6^ and for other coronaviruses^21^, which are ranging from 50 to 150 nm. By TEM, dehydrated particles show diameters distribution in the range from 40 to 95 nm (mean 61 nm ± 10 nm) , in good accordance with AFM measurement of on SARS-CoV-2 fixed with 5% glutaraldehyde^18^ and the known ≈ 25% shrinkage of samples with the dehydration procedure used here for TEM sample preparation^23^.

**Figure 2:**
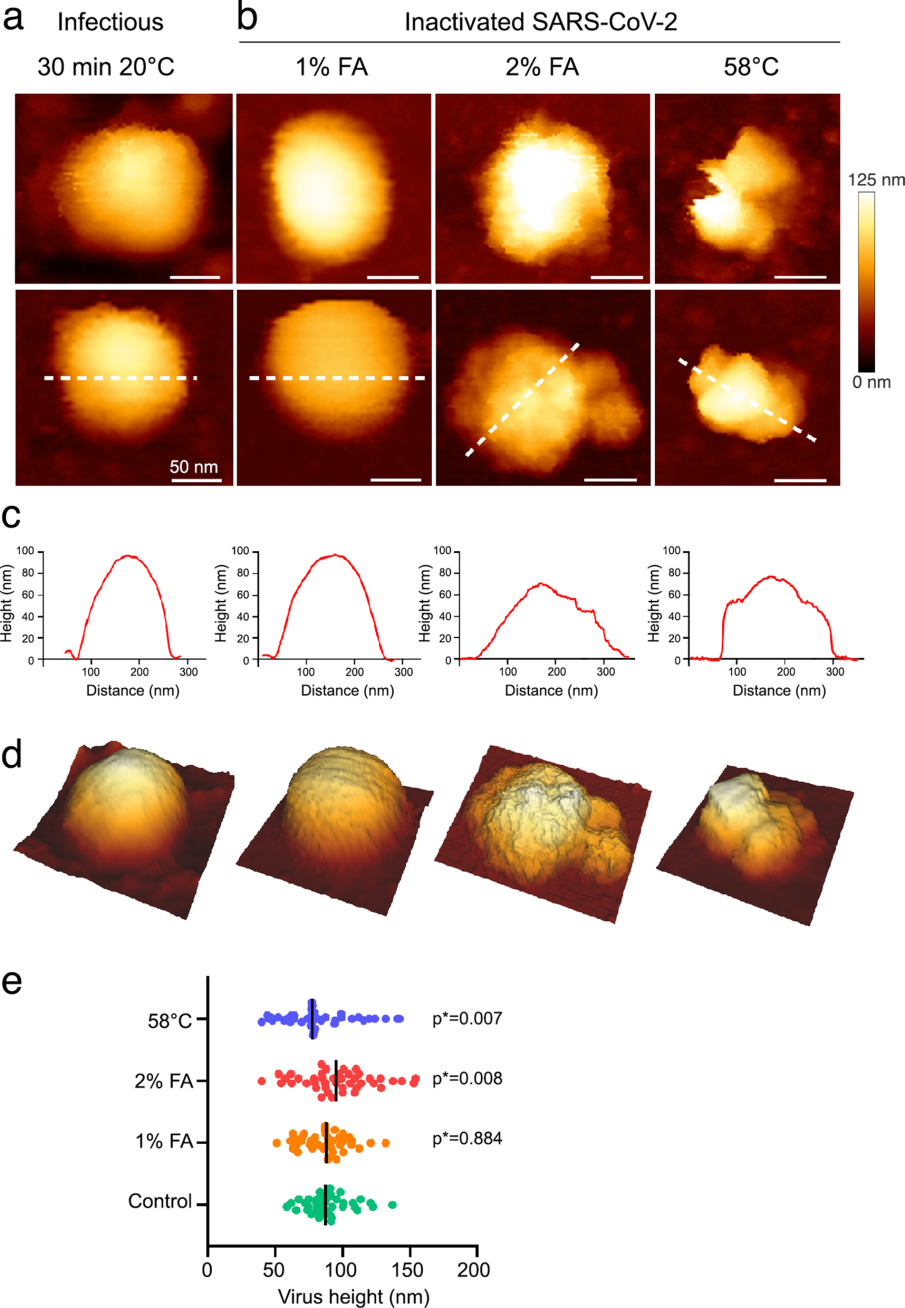
SARS-CoV-2 inactivation monitored by topographic imaging using QI mode AFM in buffer. **(a**) Native infectious, control SARS-CoV-2 sample kept for 30 min at room temperature. (**b**) Virus particles inactivated with 1% and 2% FA for 30 min at 20 °C, or incubated at 58 °C for 30 min. Scale bars are 50 nm. The color gradient for Z scale is the same for all AFM panels. (**c**) Topographical profile plots measured along the dotted lines in the lower panel in (**a**). (**d**) 3D projection of the particles in the lower panel (**a**). (**e**) Distribution of the topographical maximal height of treated and untreated SARS-CoV-2 particles (control n = 39; 1% FA n = 40; 2% FA n = 43; 58 °C n = 39). P values are indicated and were computed by using two-sided independent t tests and comparing the results to those for the untreated sample.

We then examined and compared virus inactivation using FA and heat, to identify a condition keeping structurally intact SARS-CoV-2 particles. Upon heat or FA treatments, followed by ultra-filtration, viral particles were analyzed directly by AFM for nanoscale morphology (compare Fig. 2a for WT with no treatment with Fig. 2b with treatments) and by plaque assays for infectivity (Table 1, Supplementary Figure S3). SARS-CoV-2 incubated at 58 °C for 30 min was successfully inactivated (Table 1), as described for other coronavirus^10,11^. AFM analysis of heated viruses showed severely damaged particles that have lost their spherical shapes (Fig. 2b). On the other hand, we observed complete inactivation of SARS-CoV-2 after incubation at 20 °C for 30 min with 0.5%, 1%, 2%, or 3.6% FA (see Table 1) evaluating infectivity by plaque assays (Supplementary Figure S3). AFM analysis of FA-treated viruses showed unaltered particles with a shape and height similar to the untreated control upon 0.5% or 1% FA treatment (Fig. 2b), while the higher percentage dramatically damaged viral particles, whose morphology is then similar to the samples heated to 58 °C, i.e. a loss of spherical shape and an altered average height (Fig. 2c–e). FA-fixation did not reveal the S glycoprotein as seen by others and the protrusions of the FA-treated virions did not correspond to S spikes^18^. As shown previously^10,13^, the FA inactivation effect was temperature-dependent and was strongly reduced at 4 °C. Indeed, SARS-CoV-2 was still infectious for a lower concentration of 0.1% FA with incubation at 4 °C (Table 1, Supplementary Figure S3). Altogether, these results show that SARS-CoV-2 at a concentration of 1–2 × 10^6^ PFU/ml is inactivated at 20 °C using 0.5% or 1% FA for 30 min at RT, without major loss of virus physical integrity. In contrary, keeping the virus at 4 °C in buffer, or up to 48 h at 20 °C, has no incidence on its infectious titer (Table 1) indicating a strong stability of SARS-CoV2 particles in buffer (Fig. 1a and Supplementary Figure S2).

**Table 1:** Efficiency of SARS-CoV-2 inactivation by FA in relation to temperature and incubation time.

Treatment	Temperature (°C)	Incubation time (h)	Virus titer after treatment (PFU/ml)
–	Stock − 80 °C	–	1–2 × 10^6^
–	4 °C	Up to one week	1–2 × 10^6^
–	20 °C	0.5 and up to 48 h	0.8–1 × 10^6^
–	58 °C	0.5	No plaques
No FA	20	0.5	1.4 × 10^6^
0.1% FA	4	4 h	200
0.1% FA	20	0.5	20
0.5% FA	20	0.5	No plaques
1% FA	20	0.5	No plaques
2% FA	20	0.5	No plaques
3.6% FA	20	0.5	No plaques

Virus titer was determined by plaque assays (see Supplementary Figure S3).

In conclusion, in front of the urgent need to perform researches on SARS-CoV-2 to fight COVID-19, the inactivation methods described here can contribute to transfer the virion from the confined laboratory to the lower biosafety class laboratory in an inactivated, non-infectious, form preserving viral morphology. We also show that a low percentage of FA treatment allows retaining the physical integrity of the particles albeit non-infectious. High-resolution AFM analysis of SARS-CoV-2 in buffer also demonstrates its ability to provide direct and fast qualitative information on infectious virus morphology and proves to be a method of choice for analyzing viral preparations with exceptional precision and rapidity.

## Materials and methods

### Virus and cell culture

VeroE6 cells (African green monkey kidney cells) were obtained from ECACC and maintained in Dulbecco’s minimal essential medium (DMEM) supplemented with 10% heat-inactivated fetal bovine serum (FBS) at 37 °C with 5% CO_2_. The strain BetaCoV/France/IDF0372/2020, was supplied by the National Reference Center for Respiratory Viruses hosted by Institut Pasteur (Paris, France) and headed by Pr. Sylvie van der Werf. The human sample from which strain BetaCoV/France/IDF0372/2020 was isolated has been provided by Dr. X. Lescure and Pr. Y. Yazdanpanah from the Bichat Hospital, Paris, France. Moreover, the BetaCoV/France/IDF0372/2020 strain was supplied through the European Virus Archive goes Global (EVAg) platform, a project that has received funding from the European Union's Horizon 2020 research and innovation program under the grant agreement No 653316. For supplementary Fig. S3 (panel d) we used a SARS-CoV-2 strain isolated from CPP Ile de France III, no. 2020-A00935-34 and CRB Collection of the University Hospital of Montpellier, France (https://www.chu-montpellier.fr). Both virus strains were propagated in VeroE6 cells with DMEM containing 2.5% FBS at 37 °C with 5% CO_2_ and were harvested 72 h post-inoculation. Virus stocks were stored at − 80 °C.

### Quantitative reverse transcription polymerase chain reaction (qRT-PCR)

RNAs from mock-infected or infected (MOI = 0.001) cell culture supernatant were extracted using the Nucleospin Dx Virus RNA purification kit (Macherey–Nagel). Then qRT-PCR was performed in triplicate as described^24^, using primers targeting the E gene of SARS-CoV-2 (E_Sarbeco-F ACAGGTACGTTAATAGTTAATAGCGT; E_Sarbeco-R ATATTGCAGCAGTACGCACACA) and Luna Universal One-Step qRT-PCR Kit (New England Biolabs) on a Roche Light Cycler 480. The calibration of the assay was performed with a nCoV-E-Sarbeco-Control Plasmid (Eurofins Genomics).

### Western blot

VeroE6 cells were infected for 2 h with SARS-CoV-2 (MOI = 0.001). 96 h post infection, cells were washed twice in PBS, detached with versen, pelleted at 250 × *g* for 6 min, and lysed in RIPA buffer. Total protein concentration was calculated using a Bradford protein assay kit. 20 µg of total cell lysates were diluted in Laemmli buffer and proteins were separated by SDS-PAGE on 8% (for S-protein) and 10% (for M- and N-protein) acrylamide gels. Gels were transferred to PVDF membrane using wet transfer with Tris–glycine-methanol buffer. Membranes were washed in TBS, blocked with 5% milk in TBS-T for 30 min and incubated overnight at 4 °C with primary antibody specific for spike (Gentex, cat# GTX632604), N-protein (Gentex, cat# GTX632269) or M-protein (Tebu, cat# 039100-401-A55), all three diluted at 1:1000 in TBS-T. After washing with 5% milk in TBS-T, the membranes were incubated with HRP conjugated anti-mouse antibodies for N and S protein, and with HRP conjugated anti-rabbit antibody for M protein for 2 h at room temperature, washed again in TBS-T, incubated with ECL reagent (Amersham cat#RPN2236) and imaged using a Chemidoc Imager (Biorad).

### Virus inactivation

All inactivation conditions were performed with a starting viral stock of 1–2 × 10^6^ plaque-forming units/ml (PFU/ml). Heat inactivation was performed by incubating 100 μl of SARS-CoV-2 stock at 58 °C for 30 min. Formaldehyde inactivation was performed by incubating 90 µl of virus supplied by 10 × FA-Hepes (0.5 M) at 4 °C or RT (20 °C) for 30 min, 2 h or up to overnight at 4 °C depending on the conditions described in Table 1. Samples of Fig. 2 and Supplementary Figure S3 (panels b,c “column washed”) were complemented with PBS up to 5 ml and washed using a centrifugal concentrator (Microsep advance 100KDa MWCO, Pall corporation) at 1000×*g* at 4 °C for 5 min. Samples were washed 4 times by filling again the concentrator with 5 ml of PBS and repeating the centrifugation. The last centrifugation was processed until 200 µl remained, the virus sample was collected and stored at 4 °C for one night before plaque assays. Samples of Supplementary Figure S3 (panel d “ultracentrifuged”) were diluted into 4 ml PBS after FA inactivation, loaded over a 20% sucrose cushion-TNE, and ultracentrifuged at 100.000×*g* for 1 h on a SW55ti Beckman rotor. Viral samples were resuspended in 20 µl DMEM and stored at −80 °C. Virus inactivation were monitored in plaque assay on a monolayer of VeroE6 cells (3.5 × 10^5^ cells/well), using 200 µl of virus solution. Samples were serially diluted and the PFU/mL values were determined using crystal violet coloration and subsequent scoring the amounts of wells displaying cytopathic effects.

### Atomic force microcopy

Freshly cleaved muscovite mica sheets (V1 grade, Ted Pella, Inc.) were glued on a glass slide, coated for 10 min at 20 °C with 0.1% poly-L-lysine (Sigma), rinsed with 3 ml of buffer A (10 mM Tris–HCl pH 7.5 and NaCl 100 mM) and dried with a N_2_ flux. A plastic O-ring (JPK Instruments) was then glued on the glass slide to assemble a small liquid cell. Virus samples were diluted four-fold in buffer A and 200 µL were deposited on the mica to allow passive virus adsorption. The liquid cell was completed with 200 µl of buffer A before imaging. Glass coverslips coated with hexamethyldisilazane (HDMS, Sigma) were prepared according to^20^ and AFM imaging was performed using a coverslip holder adapted to the AFM (Bruker) using the same procedure as for poly-l-Lysine coated mica. AFM imaging was performed at room temperature on a NanoWizard IV atomic force microscope (JPK Instruments-Bruker, Berlin, Germany) mounted on an inverted optical microscope (Nikon Instruments, Japan) and operating in a BSL3 laboratory. The modifications of the Bio-AFM included an additional sealing of the AFM scanner head, protection of electronic parts, and a specific machining (smoothing) of the metallic elements (object holder, cantilever) avoiding sharp edges. Topographic imaging was performed in quantitative imaging (QI) mode, which is a force-curve-based imaging mode, using BL-AC40TS-C2 cantilevers (mean cantilever spring constant k_cant_ = 0.09 N/m, Olympus). Before each experiment, the sensitivity and spring constant (thermal noise method) of the cantilever were calibrated. The applied force was kept at 150 pN and a constant approach/retract speed of 10 µm/s (z range of 100 nm). For Fig. 1c, the applied force was raised to 1 nN and a constant approach/retract speed of 1 µm/s. Images were flattened with a polynomial/histogram line-fit with the JPK data processing software. Low-pass Gaussian and/or median filtering was applied to remove minor noise from the images. The Z-color scale in all images is given as relative after processing, with the offset being kept the same within each of the figures to emphasize the structural features. Particle height analysis was carried out using the height (measured) channel of the QI mode, which corresponds to the height at 80% of the setpoint force determined on the reference F-D curve. Height was calculated as the topographical maximal central height on each virion using the section tool of the analysis software, and the histogram tool of the z channel values using a 300 × 300 nm area for each particle.

### Transmission electron microcopy

VeroE6 cells were infected with 1 × 10^6^ PFU of SARS-CoV-2 for 24 h. Cells were fixed with 2.5% (v/v) glutaraldehyde in PHEM buffer and post-fixed in osmium tetroxide 1% / K_4_Fe(CN)_6_ 0,8%, at room temperature for 1 h for each treatment. The samples were then dehydrated in successive ethanol baths (50/70/90/100%) and infiltrated with propylene oxide/EMbed812 mixes before embedding. 70 nm ultrathin cuts were made on a PTXL ultramicrotome (RMC, France), stained with OTE/lead citrate and observed on a Tecnai G2 F20 (200 kV, FEG) TEM at the Electron Microscopy Facility MRI-COMET, INM, Plate-Forme Montpellier RIO Imaging, Biocampus, Montpellier.

## Supplementary Information

Supplementary Figures.
