# Supplementary material for: Atomic force microscopy analysis of native infectious and inactivated SARS-CoV-2 virions
Source: Sci Rep. 2021 Jun 4;11:11885. doi: 10.1038/s41598-021-91371-4 (PMC8178396; doi:10.1038/s41598-021-91371-4)
Supplement: Supplementary file 1 — Supplementary Figures. [file 41598_2021_91371_MOESM1_ESM.pdf]

Atomic Force Microscopy analysis of native infectious and inactivated SARS-CoV-2 virions

Sébastien Lyonnais[1,*], Mathilde Hénaut[1], Aymeric Neyret[1], Peggy Merida[2], Chantal Cazevieille[3], Nathalie Gros[1], Christine Chable-Bessia[1] and Delphine Muriaux[1,2 *]

Affiliations

[1]CEMIPAI, University of Montpellier, UAR3725 CNRS, Montpellier, France

[2]Institute of Research in Infectiology of Montpellier (IRIM), University of Montpellier, UMR9004 CNRS, Montpellier, France

[3]Université de Montpellier, Institut des Neurosciences de Montpellier (INM), Montpellier, France

*Corresponding authors: sebastien.lyonnais@cemipai.cnrs.fr, delphine.muriaux@cemipai.cnrs.fr

SUPPLEMENTARY INFORMATION

Supplementary Figures

Figure S1. SARS-CoV-2 identification

Figure S2. AFM images of native infectious SARS-CoV-2

Figure S3. Native and inactivated SARS-CoV-2 titration followed by plaque assays

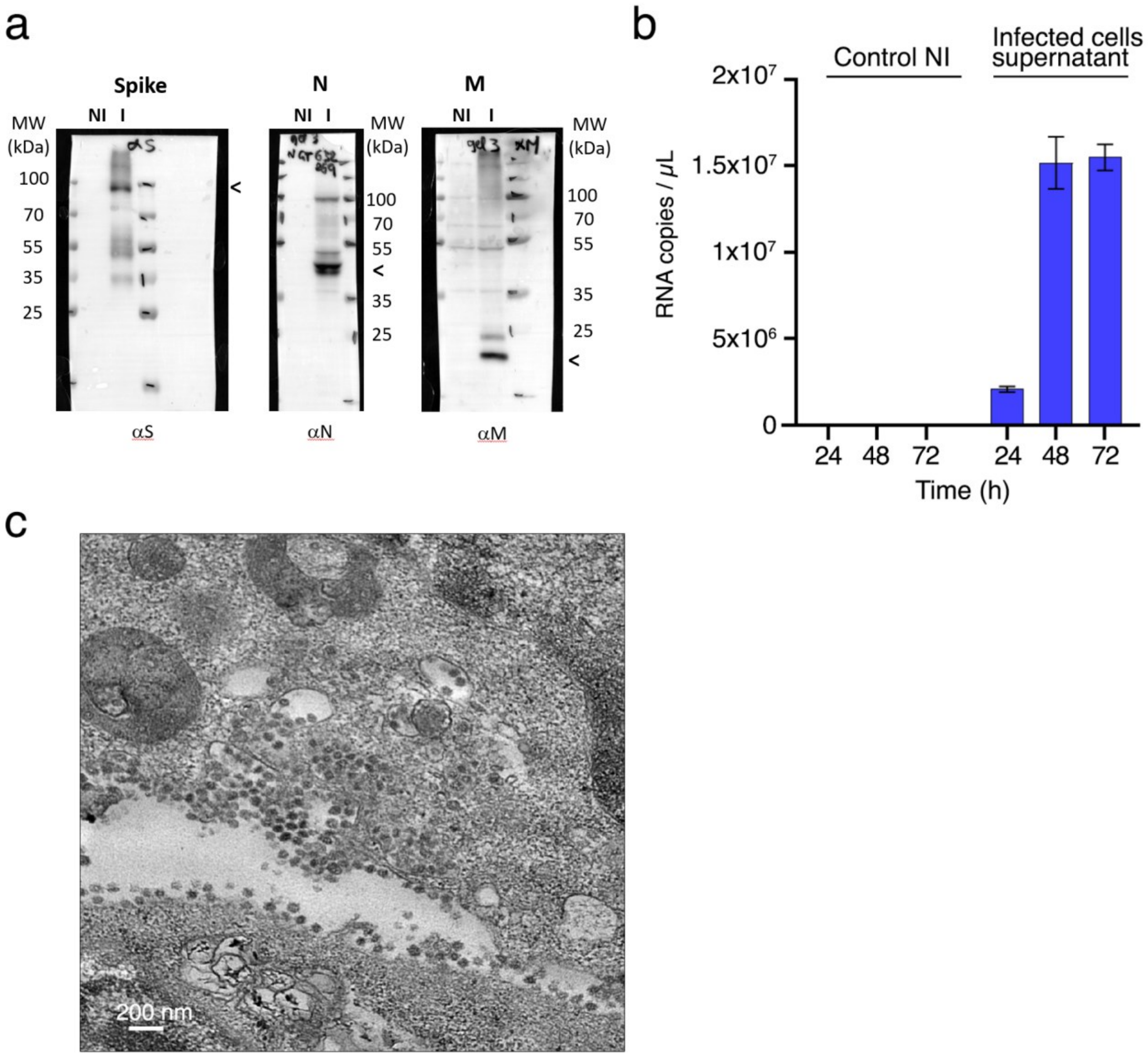

Supplementary Figure S1. SARS-CoV-2 identification. (**a**) Western Blot analysis of SARS-CoV-2 S, N and M proteins in lysates of infected (I) and non infected (NI) VeroE6 cells. (**b**) SARS-CoV-2 production in the cell supernatant followed by qRT-PCR targeting the E gene. (**c**) An example of TEM image of SARS-CoV-2 infected VeroE6 cells for 24h (MOI=0.1)

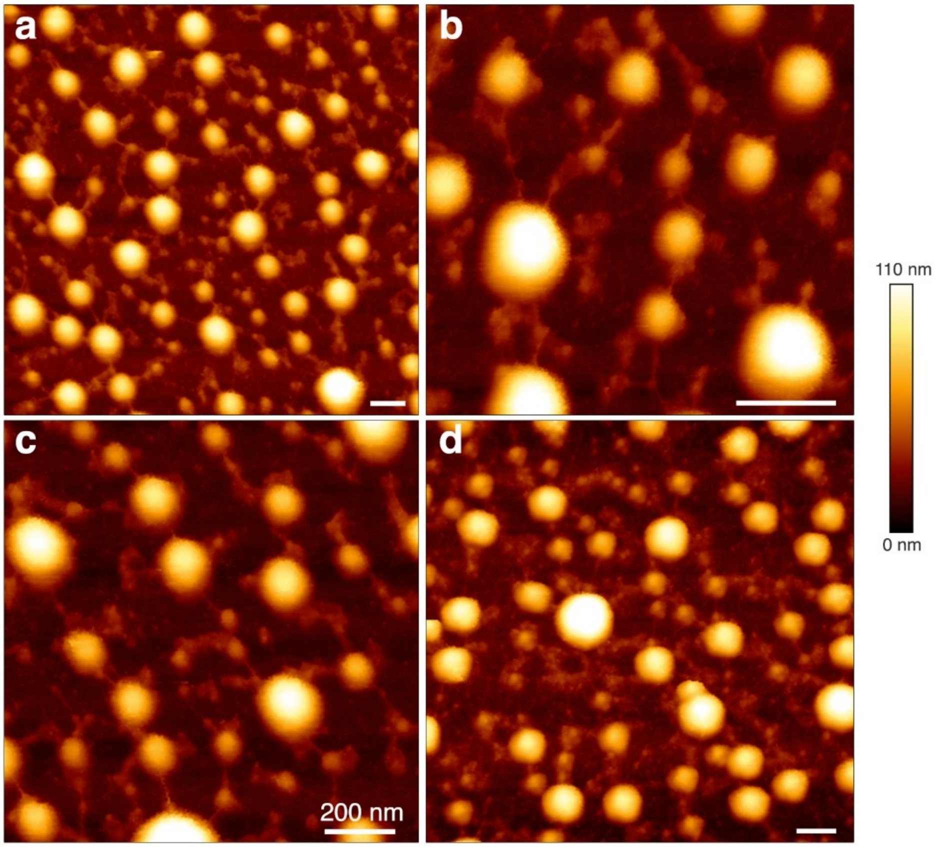

Supplementary Figure S2. AFM images of native infectious SARS-CoV-2 found embedded in a network of filamentous structures. (a-d) AFM topographic images of infectious native SARS-CoV-2 from cell supernatant directly adsorbed on a mica surface using QI mode AFM in buffer. Scale bars in all panels: 200 nm.

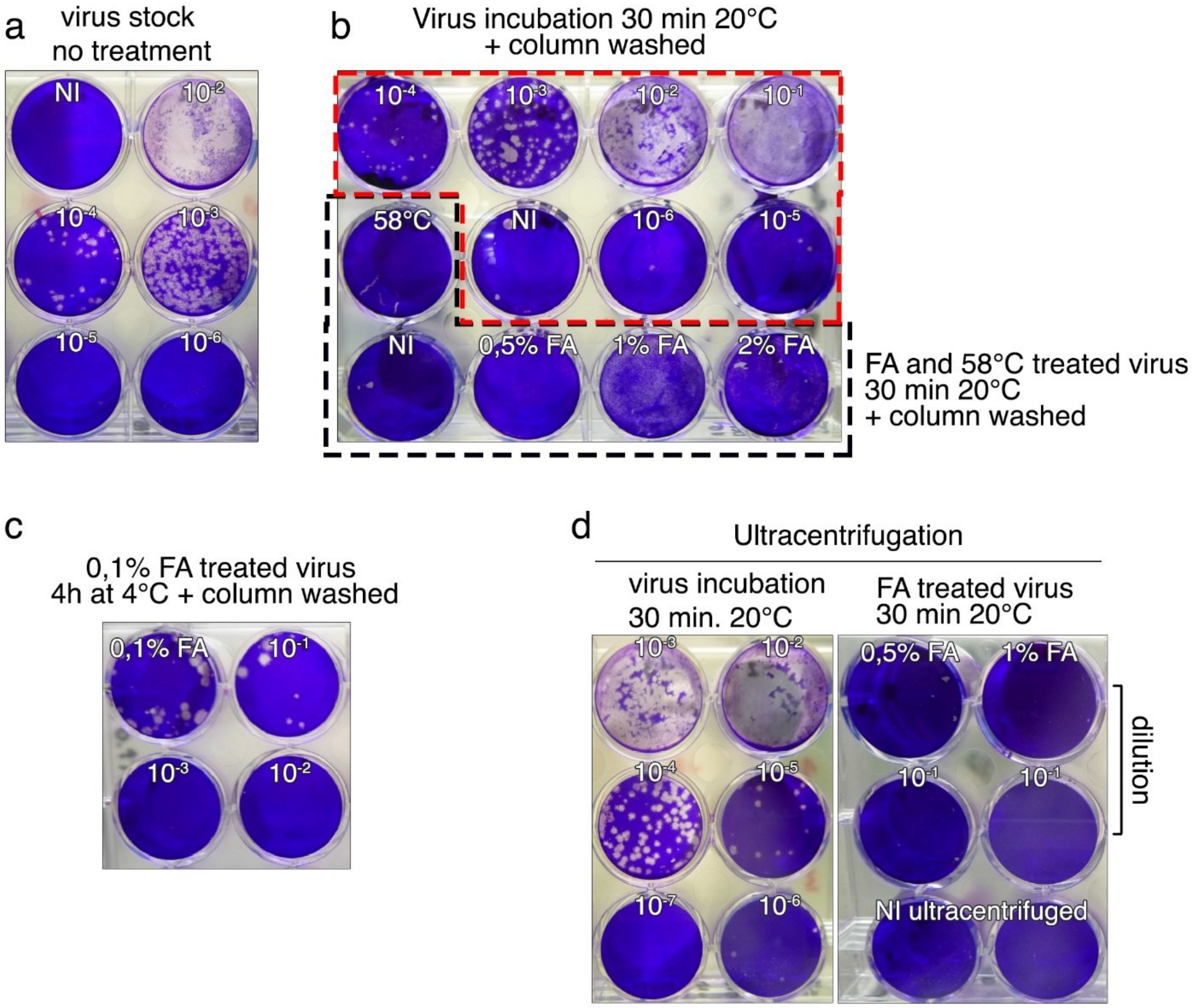

Supplementary Figure S3. Native and inactivated SARS-CoV-2 titration followed by plaque assays (**a**) Viral stock titration by 10-fold dilutions of SARS-CoV-2 specimen from 10^{-2} to 10^{-6}, as well as non-infected cells supernatant (NI) as a negative control. Plaques can be distinguished from the crystal violet monolayer and used for the calculation of virus titers in Plaque Formation Unit/mL (PFU/mL) (**b**) Virus titration after incubation of SARS-CoV-2 specimen for 30 min. at 20°C followed by 5 washes using centrifugal concentrator (red area). Undiluted virus samples incubated for 30 min. at 58°C or 30 min. at 20°C in presence of 0.5%, 1% or 2% Formaldehyde (FA), followed by 5 washes using centrifugal concentrator (black area). (**c**) Virus titration after virus incubation for 4h at 4°C in presence of 0.1% FA. (**d**) Virus recovered from ultracentrifugation after incubation for 30 min. at 20°C in absence (left panel) or in presence of 0.5% or 1% FA.
